# *ℏ*^2^ Corrections to Semiclassical Transmission Coefficients

**DOI:** 10.1021/acs.jpca.4c00452

**Published:** 2024-04-17

**Authors:** Sameernandan Upadhyayula, Eli Pollak

**Affiliations:** Chemical and Biological Physics Department, Weizmann Institute of Science, Rehovot 76100, Israel

## Abstract

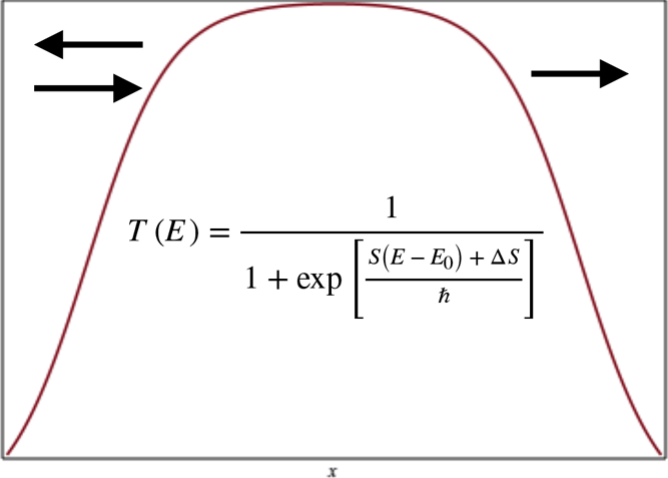

The uniform semiclassical expression for the energy-dependent transmission probability through a barrier has been a staple of reaction rate theory for almost 90 years. Yet, when using the classical Euclidean action, the transmission probability is identical to 1/2 when the energy equals the barrier height since the Euclidean action vanishes at this energy. This result is generally incorrect. It also leads to an inaccurate estimate of the leading order term in an *ℏ*^2*n*^ expansion of the thermal transmission coefficient. The central result of this paper is that adding an *ℏ*^2^ dependent correction to the uniform semiclassical expression, whether as a constant action or as a shift in the energy scale, not only corrects this inaccuracy but also leads to a theory that is more accurate than the previous one for almost any energy. Shifting the energy scale is a generalization of the vibrational perturbation theory 2 (VPT2) and is much more accurate than the “standard” VPT2 theory, especially when the potential is asymmetric. Shifting the action by a constant is a generalization of a result obtained by Yasumori and Fueki (YF) only for the Eckart barrier. The resulting modified VPT2 and YF semiclassical theories are applied to the symmetric and asymmetric Eckart barrier, a Gaussian barrier, and a tanh barrier. The one-dimensional theories are also generalized to many-dimensional systems. Their effect on the thermal instanton theory is discussed.

## 1 Introduction

Almost nine decades ago, Kemble^1^ derived a semiclassical expression for the energy-dependent transmission probability through a potential barrier:
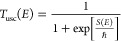
1.1where *E* is the energy relative to reactants, the barrier energy is denoted as *V*^‡^, and *S*(*E*) is the Euclidean action on the upside-down potential energy surface:
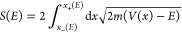
1.2*x*_±_(*E*) are the turning points defined by the equation *V*(*x*) = *E*. Equation 1.2 is valid for energies below and above the barrier, but unless the action is known analytically as for the Eckart barrier, above the barrier the turning point becomes complex so that its computation is not as straightforward.^2−4^

This uniform semiclassical expression has been used extensively for calculating thermal transmission coefficients for 1-D systems such as Eckart barriers and many other potential energy models.^5−7^ It turns out that in one dimension, Kemble’s expression is the most accurate semiclassical expression available for thermal transmission coefficients, better than classical Wigner dynamics,^8−16^ centroid molecular dynamics,^17^ and ring polymer molecular dynamics,^18^ especially in the high temperature limit.^9^

The uniform expression has been used in a multidimensional context, which enables the computation of transmission probabilities, using only up to fourth-order derivatives of the potential around the barrier top. Miller and co-workers showed how it may be used for the computation of cumulative reaction probabilities in multidimensional systems within the framework of vibrational perturbation theory (VPT).^20,21^ The fact that only up to fourth-order derivatives are needed implies that the resulting theory is very convenient for use with ab initio quantum chemistry computations.

There was, though, something missing in the theory. The second-order vibrational perturbation theory was used originally to evaluate the energy levels of molecules in terms of products of  where *n*_*j*_ is the quantum number of mode *j*.^19^ Setting all *n*_*j*_’s to zero gives the ground state energy. However, VPT gives an additional constant term *E*_0_, which was not included in the original development of refs (20,21) but was added later on by Barker and co-workers to give what is known today as the VPT2 theory.^22^ One of the difficulties with VPT2 theory is that it is not very precise for deep tunneling, especially when the potential is asymmetric.^23^

The uniform semiclassical expression is central to the multidimensional microcanonical instanton rate theory which has been explored by Richardson and co-workers.^7,24^ Here, the instanton refers to a periodic orbit on the inverted potential energy surface. Numerical algorithms for its computation have been derived in refs (7,24−27).

Yet, the microcanonical instanton rate theory also has deficiencies, especially when the energy is above the barrier height, where one has to resort to the parabolic barrier estimate,^28^ which is not very accurate since it does not account for nonlinearities and so is incorrect even when considering the leading order term in *ℏ*^2^.^29−31^

Kemble’s expression is also fundamental to our recently developed uniform instanton rate theory,^32^ which removed the divergence inherent to the instanton expression for the thermal rate at the “crossover temperature”. Using a steepest descent evaluation based on Kemble’s expression, we showed that there is no divergence at all. The one-dimensional uniform instanton theory was generalized to multidimensional systems in ref (34).

Yet, there remains a fundamental problem in Kemble’s expression. When the energy *E* equals the barrier energy *V*^‡^, the action *S*(*E*) = 0 and the resulting transmission probability is 1/2 (the half point) independent of the form of the barrier. This is clearly not the case, as may be seen from analyzing the symmetric and asymmetric Eckart barriers as well as the square barrier.

All of this implies that any improvement to the uniform semiclassical expression would be beneficial. Uniquely for the Eckart barrier, Yasumori and Fueki,^36^ using an earlier idea of Eckart^35^ showed that replacing all cosh(*x*) terms in Eckart’s exact transmission probability expression with exp(*x*)/2 leads to the Kemble form; however, the action is modified by an additional term, which goes as *ℏ*^2^. The Yasumori Fueki approximation is much more accurate for the Eckart barrier than Kemble’s expression using the Euclidean classical action.

Combining the observations coming from both the VPT2 theory and the Yasumori Fueki correction implies that there might well be a simple way to improve upon Kemble’s original expression, by including *ℏ* dependent terms in the action. This is the topic of this paper. At the outset, we note that if one knows the exact energy-dependent transmission probability *T*(*E*), one may always invert eq 1.1 to obtain the “exact quantum action” through the relation:
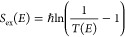
1.3This might seem to be useless; however, it does imply that there exists an action that would improve the semiclassical estimate and that this improved action would necessarily depend on *ℏ*.

In this paper, we present two differing improved expressions. One, which we term the modified VPT2 expression, involves a simple shift of the energy of the Euclidean action:
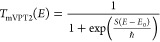
1.4where *E*_0_ is as yet an undetermined energy parameter. The second expression is guided by the Yasumori Fueki expression for the Eckart barrier, whereby the action is shifted by a constant-energy-independent term Δ*S*:
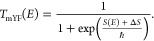
1.5 To derive expressions for these parameters, we will demand that when thermally averaged, the resulting quantum transmission coefficient will be exact at least up to order *ℏ*^2^.

One notes from both of these expressions that these modifications move the half point of the transmission probability. If *E*_0_ < 0 then the half-point for *T*_mVPT2_ will occur at an energy lower than the barrier energy since the action will vanish when *E* = *V*^‡^ + *E*_0_ < *V*^‡^. Conversely, if it is positive, then the half-point will occur later. The same happens with *T*_mYF_. When Δ*S* is negative (positive) the half point of *T*_mYF_ moves to an energy that is lower (greater) than the barrier energy. We will see that both expressions significantly improve the resulting transmission probabilities.

The one-dimensional theory is presented in Section 2 and implemented in Section 3 for the symmetric and asymmetric Eckart barriers, a symmetric Gaussian barrier, and the tanh potential model introduced in ref (37). The theory is generalized to many-dimensional systems in Section 4. We end with a discussion of the relative merits of the mVPT2 and mYF theories, their implications for thermal instanton rate theory, and improving the corrections to Kemble’s expression systematically.

## 2 *ℏ*^2^ Corrections to the Action

### 2.1 The High-Temperature Limit

The central theoretical development needed is to derive the leading order quantum corrections to the thermal rate when using the modified theories as in eqs 1.4 and 1.5. Without loss of generality, we consider a potential barrier *V*(*q*) such that *V* (*q* → –*∞* = 0), a barrier height *V*^‡^ and an imaginary frequency ω^‡^ at the barrier top. The product region is identified by the energy −*V*_*∞*_ where *V*_*∞*_ ≥ 0. The thermal transmission coefficient is defined as the ratio of the quantum to the classical thermally averaged transmission probabilities:
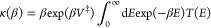
2.1where *T*(*E*) is the exact energy-dependent transmission probability and  is the inverse temperature (*k*_B_ is the Boltzmann constant). Pollak and Cao showed that the leading order correction to the thermal transmission probability in terms of the inverse temperature and *ℏ* dependent parameter:
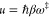
2.2is^31^
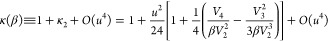
2.3where *V*_*n*_ is the *n*-th derivative of the potential at the barrier top.

In this high-temperature limit, the main contribution to the thermal transmission coefficient comes from energies that are in the vicinity of the barrier height or greater. To obtain the leading order *ℏ*^2^ correction based on the Kemble form it is then sufficient to expand the action to the second order:
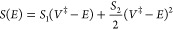
2.4where *S*_*n*_ is the *n*-th derivative of the action with respect to the energy at the barrier energy. The first and second derivatives are well-known^20^
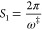
2.5and
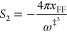
2.6with
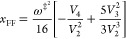
2.7

### 2.2 *ℏ*^2^ Expansion for the Uniform Semiclassical Thermal Rate

The expansion of the uniform semiclassical expression obtained from thermal averaging is given in eq A.13 of the Appendix by setting the two parameters Δ*S*_2_ = Δ*E* = 0 and using eqs 2.6 and 2.7:
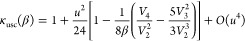
2.8This is not the same as the exact result given in eq 2.3. Usually, the fourth-order derivative is positive so that the uniform estimate as given in eq 2.8 will be *less* than the parabolic barrier estimate, while in reality, as seen from eq 2.3 the exact transmission coefficient will be *larger*. This is a reflection of the incorrect half-point of the uniform theory. When *V*_4_ ≥ 0, the half point will be found at an energy that is lower than the barrier height and therefore the exact transmission coefficient is larger than predicted from the uniform expression of Kemble and the parabolic barrier limit of 1 + *u*^2^/24.

### 2.3 A Modified VPT2 Theory

Setting the action coefficient Δ*S*_2_ = 0 in eq A.13 gives the leading order expansion for the modified VPT2 transmission coefficient defined in eq 1.4 as
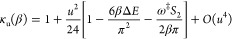
2.9We are now in the position of being able to determine the energy shift *E*_0_ = *ℏ*^2^Δ*E* by equating this result with the exact expansion as given in eq 2.3. One readily finds
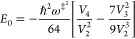
2.10and this is precisely the zero point energy shift that appears in VPT2 theory.^23^

This is not an accident. As shown in ref (31), VPT2 theory gives the correct leading order *ℏ*^2^ term for the transmission coefficient, so, by construction, one should expect this result. However, there is a fundamental difference between the modified VPT2 theory in eq 1.4 and the “standard” VPT2 theory. In the latter case, the energy action relation is expanded to the second order, and this imposes that the VPT2 action is given by the expression:
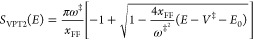
2.11In contrast, in eq 1.4, the exact Euclidean action is shifted in energy by the constant factor *E*_0_. The difference is especially significant when the temperature is low and the energy region contributing mostly to the thermal transmission factor, the instanton energy, is far below the barrier energy. There is then no obvious reason why an expansion of the potential only to the fourth order about the barrier top should give the correct action, as discussed below when considering specific examples, it does not. We will see that this is the major source of error in the VPT2 theory. The modified theory of eq 1.4 corrects this and gives a much-improved estimate.

There is another advantage of the modified theory. The VPT2 action energy relationship of eq 2.11 makes sense only if
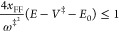
2.12If *x*_FF_ is negative, as is the case when *V*_4_ ≥ 0, then there would be a lower cutoff on the energy
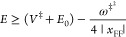
2.13such that for lower energies in the deep tunneling region the action becomes complex, and this would make little sense. The mVPT2 theory does not have this issue, as the Euclidean action is real and positive for all energies below the barrier height. Conversely, if *V*_4_ ≤ 0 then *x*_FF_ ≥ 0, and this will impose an upper bound on the energy for which the VPT2 theory is valid
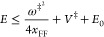
2.14It will also impose a lower bound *E* ≥ *E*_0_ for which the mVPT2 expression is valid since the Euclidean action *S*(*E*) is not well defined for negative energies.

The modified VPT2 theory, as given in eq 1.4, is not yet complete. If one knows the action analytically, then it is complete, as, for example, in the case of an Eckart barrier. In general, below the barrier, the action function would be computed numerically, but for above-barrier energies, the computation would not be simple, especially when doing it on the fly, since one needs to determine complex turning points. If *x*_FF_ ≤ 0, which is generally the case, then for energies above the barrier the VPT2 action as given in eq 2.11 remains valid so that the modified VPT2 theory in this case will be defined as
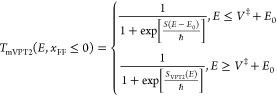
2.15and in this case, the transmission coefficient will be continuous about the half-point *E* = *V*^‡^ + *E*_0_. If *x*_FF_ ≥ 0, then the VPT2 action will become invalid and we replace it such that
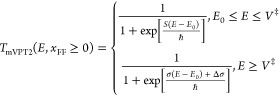
2.16where σ(*E*) is the second-order expansion of the action about the barrier energy *V*^‡^:
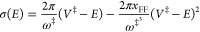
2.17and Δσ is a constant, which assures continuity of the transmission coefficient at *E* = *V*^‡^:
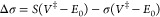
2.18and is of the order of *ℏ*^4^ so it will be very small in practice.

Equations 2.15 and 2.16 are the central results for this subsection. They define a uniform semiclassical theory with a small quantum correction to the action, assured to give the exact leading order correction term to the thermal transmission probability.

### 2.4 Modified Yasumori-Fueki Theory

We note from eq A.13 that by setting Δ*E* = 0 the thermal transmission coefficient up to order *u*^2^ is
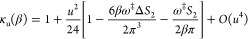
2.19Equating this with the exact result (eq 2.3) implies that
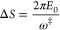
2.20Implementing the modified Yasumori-Fueki theory at energies below the barrier height is then straightforward. One evaluates the Euclidean action and adds to it the correction term Δ*S* which is of the order of *ℏ*^2^. If the action function is known analytically as in the Eckart barrier, then the theory is complete. However, if the action must be determined numerically, and this is the generic case, then as in the mVPT2 theory, one needs a “good” form for the action, especially at above barrier energies.

When *V*_4_ ≥ 0 we know that *E*_0_ ≤ 0 so that at the energy *V*^‡^ + *E*_0_ the Euclidean action is still well-defined. At this energy
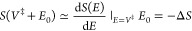
2.21where the last equality follows from eqs 2.5 and 2.20. This implies that
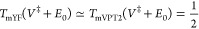
2.22Therefore, it is possible to combine the mYF with the mVPT2 result so that
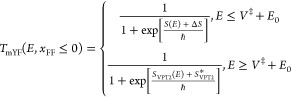
2.23where *S*_VPT2_^*^ is defined to ensure that the transmission coefficient is continuous at *E* = *V*^‡^ + *E*_0_:
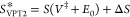
2.24Note that in view of eqs 2.21 and 2.22, *S*_VPT2_^*^ is of the order of *ℏ*^4^, so it will be typically almost negligible.

If *x*_FF_ ≥ 0, then the Euclidean action is well defined through the periodic orbit on the inverse potential energy only up to the energy *E* = *V*^‡^ ≤ *V*^‡^ + *E*_0_. Therefore, in this case, the modified YF transmission coefficient will be defined as
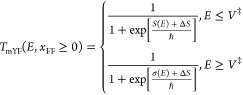
2.25and it is continuous at *E* = *V*^‡^ with no further modification. In contrast to the mVPT2 theory, *T*_mYF_(*E*) is well-defined for all energies.

## 3 Numerical Applications

In this section, we will apply the modified theories for some one-dimensional model potentials: the symmetric Eckart potential, the asymmetric Eckart potential, a Gaussian potential, and the tanh potential defined in ref (37).

### 3.1 Symmetric Eckart Barrier

The Hamiltonian for the symmetric Eckart potential is
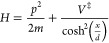
3.1The barrier frequency is . The exact energy-dependent transmission probability^35^ is
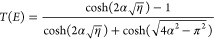
3.2where α = 2π*V*^‡^/(*ℏ*ω^‡^) is a measure of the width of the barrier and  is the reduced energy.

The Euclidean action for this system is^21,23,26^
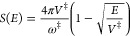
3.3The VPT2 action is somewhat different
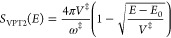
3.4where
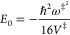
3.5so that in this symmetric Eckart barrier case, the modified VPT2 theory is identical to the original VPT2 theory.

The standard Yasumori action^36^ is slightly different from the VPT2 action
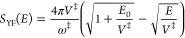
3.6 The modified YF action is
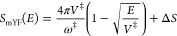
3.7and one notes that it is just the leading order term in the expansion of the YF action (eq 3.6) in terms of *E*_0_/*V*^‡^.

All the transmission coefficients are determined by the single dimensionless parameter . Following previous computations^32,38,39^ we choose it to be . At this reduced barrier height, the shift energy *E*_0_ = −0.125 and Δ*S* = −0.206 so that the magnitude of *E*_0_ is much less than the barrier energy. Results for the thermal transmission factors obtained by numerical integration over energy are given in Table 1. Columns 2–6 correspond to the exact thermal transmission factor (eq 3.2), the modified Yasumori–Fueki transmission factor using the action as in eq 3.7, the VPT2 transmission factor using the action function as in eq 3.4, the Yasumori–Fueki transmission factor using the action as in eq 3.6 and the uniform semiclassical (usc) transmission factor using the action as in eq 3.3.

**Table 1:** Thermal Transmission Coefficients for the Symmetric Eckart Barrier

*ℏ*βω^‡^	*T*_exact_	*T*_mYF_	*T*_mVPT2_	*T*_YF_	*T*_usc_
0.5	1.021	1.021	1.021	1.021	1.004
1.0	1.064	1.064	1.065	1.064	1.030
1.5	1.129	1.129	1.132	1.129	1.077
2	1.224	1.224	1.227	1.224	1.149
3	1.525	1.524	1.535	1.525	1.391
4	2.071	2.070	2.097	2.071	1.839
6	5.198	5.195	5.372	5.198	4.414
8	21.769	21.752	23.352	21.769	17.973
10	161.905	161.782	183.317	161.922	132.174
12	1973.338	1972.397	2379.648	1974.144	1606.829
14	34,057.358	34,063.686	43,769.402	34,093.945	27,735.744
16	7.404 × 10^5^	7.414 × 10^5^	1.014 × 10^6^	7.420 × 10^5^	6.036 × 10^5^
18	1.882 × 10^7^	1.888 × 10^7^	2.747 × 10^7^	1.889 × 10^7^	1.537 × 10^7^
20	5.344 × 10^8^	5.373 × 10^8^	8.294 × 10^8^	5.377 × 10^8^	4.374 × 10^8^

It is striking that the Yasumori–Fueki and modified Yasumori–Fueki estimates are almost identical and very close to the exact result, covering 8 orders of magnitude, and are larger and more accurate than the usc estimate at all temperatures. This reflects the fact that in the usc estimate the half point energy is *V*^‡^, which is too large. Also notable is that all estimates (apart from the usc estimate) are quantitative in the high-temperature region, that is, for *u* ≤ 2. This is because they are constructed to give the exact transmission coefficient in this temperature range.

Further insight may be obtained by considering the energy action relations. One finds that
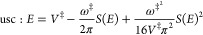
3.8
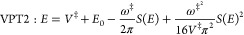
3.9
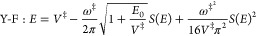
3.10In all cases, one has a quadratic energy action relation. The difference between the VPT2 and the Y–F relation is that for the VPT2 case, the energy is shifted by *E*_0_ while in the Y–F case, one may say that the barrier frequency is quantum-corrected.

### 3.2 Asymmetric Eckart Barrier

The Hamiltonian for the asymmetric Eckart barrier is
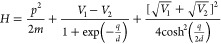
3.11where *V*_1_ is the barrier height *V*_1_ – *V*_2_ is the exoergicity of the barrier and *d* is the length scale. The barrier top is found at . The Euclidean action as a function of energy^26^ is
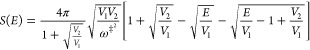
3.12

The exact quantum energy-dependent transmission probability for the asymmetric case is
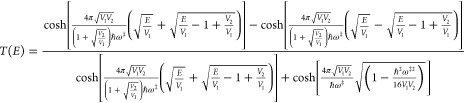
3.13As suggested by Eckart^35^ and implemented by Yasumori and Fueki^36^ an excellent approximation to the exact transmission coefficient is obtained by replacing all the cosh functions with their positive exponent components. This leads to the Y–F approximation for the transmission probability for which the action is
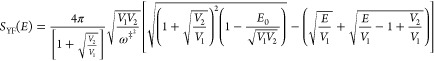
3.14Note that typically  so that one can expand the square root to the leading order in *E*_0_ to obtain the mYF action, which is
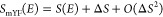
3.15

Using the same parameters for the asymmetric case, as in refs (32,38),  and , we obtain *E*_0_ = −0.0278 and Δ*S* = −0.0685 and the results given in Table 2. The magnitude of *E*_0_ is much smaller than the barrier height, as expected, yet it is significant. As in the symmetric case, at high temperatures (small *u*), all rates are almost identical except for the “standard” usc approximation, which, as expected, is too low. The agreement of all other approximations with the exact result reflects the fact that the approximations have been constructed to be exact in this high-temperature limit. As the temperature is lowered (*u* becomes larger) one moves into the deep tunneling regime, and both VPT2 and mVPT2 estimates become higher than the exact result. However, the mVPT2 theory is superior to the standard VPT2 theory. This is because the mVPT2 theory uses the energy-shifted classical action function for energies below the barrier and does not assume the quadratic energy action relationship as in the VPT2 theory. In the asymmetric case, one cannot express the energy as a quadratic function of the Euclidean action. The YF and mYF estimates for the thermal transmission coefficient are almost identical for the whole range of *u* values considered and as in the symmetric case are superior to the usc approximation even in the low-temperature deep tunneling regime. Their error is at worst ∼3%. The mYF theory has the advantage that it can be generalized to any potential barrier, even if one does not know the analytical dependence.

**Table 2:** Thermal Transmission Coefficients for the Asymmetric Eckart Barrier

*ℏ*βω^‡^	*T*_exact_	*T*_mYF_	*T*_mVPT2_	*T*_YF_	*T*_VPT2_	*T*_usc_
0.5	1.014	1.014	1.014	1.014	1.014	1.006
1.0	1.050	1.050	1.050	1.050	1.050	1.033
1.5	1.109	1.109	1.109	1.109	1.109	1.082
2	1.195	1.195	1.196	1.195	1.197	1.157
3	1.480	1.480	1.484	1.480	1.487	1.412
4	2.015	2.015	2.025	2.015	2.037	1.896
6	5.322	5.322	5.401	5.322	5.583	4.895
8	26.097	26.095	26.989	26.097	31.025	23.680
10	251.558	251.570	267.471	251.597	382.373	227.231
12	4067.815	4069.390	4460.496	4069.839	8335.739	3672.367
14	90,557.643	90,650.342	1.024 · 10^5^	90,660.343	2.459 × 10^5^	81,795.631
16	2.445 × 10^6^	2.450 × 10^6^	2.848 × 10^6^	2.450 × 10^6^	8.484 × 10^6^	2.210 × 10^6^
18	7.466 × 10^7^	7.490 × 10^7^	8.959 × 10^7^	7.491 × 10^7^	3.195 × 10^8^	6.759 × 10^7^
20	2.476 × 10^9^	2.489 × 10^9^	3.069 × 10^9^	2.489 × 10^9^	1.268 × 10^10^	2.245 × 10^9^

### 3.3 Gaussian Barrier

As noted in the previous section, the mVPT2 and mYF expressions change when one does not have an analytical expression for the Euclidean action for the whole energy range. To see what happens in such a case, we present here the results for a Gaussian barrier:
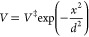
3.16where *V*^‡^ is the barrier height and *d* is the length scale. The barrier frequency is . The thermal transmission coefficients are calculated using the same parameters as in the symmetric Eckart barrier, i.e., . The Euclidean action is computed numerically. The turning points are known analytically so that the remaining one-dimensional integration is performed by using the Cuhre integration algorithm within the Maple software. The numerically exact energy-dependent transmission probability for the Gaussian barrier is calculated by solving the Schrödinger equation using Maple, with an error tolerance of ∼1 × 10^–6^. The thermal transmission factor is then obtained by numerical integration over the energy. In the units defined above, the fourth derivative of the potential *V*_4_ = 12*V*^‡^ is positive so that *x*_FF_ < 0 and *E*_0_ < 0. We find that *E*_0_ = −0.09375 ≪ *V*^‡^ = 72/π^2^, Δ*S* = −0.1542 (see eq 2.24), and *S*_VPT2_^*^ = 0.000374. Although the magnitude of *E*_0_ is small compared to the barrier height, it is not negligible, shifting the midpoint of the transmission probability to energies lower than the barrier energy in both the mVPT2 and mYF results. This also underlies the fact that the resulting thermal transmission probabilities are larger than the usc estimate.

The results are shown in Table 3. Here, we do not tabulate the usc estimate since the action needs to be determined from the complex turning points when the temperature is high. As for the Eckart barrier, at high temperatures, all other approximations give practically identical results. Things become interesting at low temperatures. The VPT2 estimate fails, it is a factor of ∼3.5 greater than the exact result at *u* = 20. Using the mVPT2 expression leads in this case to an overestimate of only ∼10%. The mYF theory is only slightly worse, with an underestimate of ∼16% when *u* = 20. This numerical example accentuates the need to modify the VPT2 theory and shows that the modification we are using leads to rather accurate estimates even in the deep tunneling regime.

**Table 3:** Thermal Transmission Coefficients for the Gaussian Barrier

*ℏ*βω^‡^	*T*_exact_	*T*_mYF_	*T*_mVPT2_	*T*_VPT2_
0.5	1.019	1.018	1.019	1.019
1.0	1.059	1.058	1.059	1.059
1.5	1.124	1.122	1.123	1.124
2	1.216	1.213	1.216	1.217
3	1.522	1.514	1.521	1.524
4	2.096	2.077	2.094	2.104
6	5.711	5.570	5.695	5.864
8	29.486	27.856	29.292	33.081
10	306.090	276.908	303.203	412.980
12	5353.378	4659.992	5328.533	9061.192
14	1.276 × 10^5^	1.083 × 10^5^	1.290 × 10^5^	2.685 × 10^5^
16	3.644 × 10^6^	3.050 × 10^6^	3.772 × 10^6^	9.292 × 10^6^
18	1.162 × 10^8^	9.691 × 10^8^	1.240 × 10^8^	3.505 × 10^8^
20	3.986 × 10^9^	3.335 × 10^9^	4.399 × 10^9^	1.393 × 10^10^

**Figure 1:**
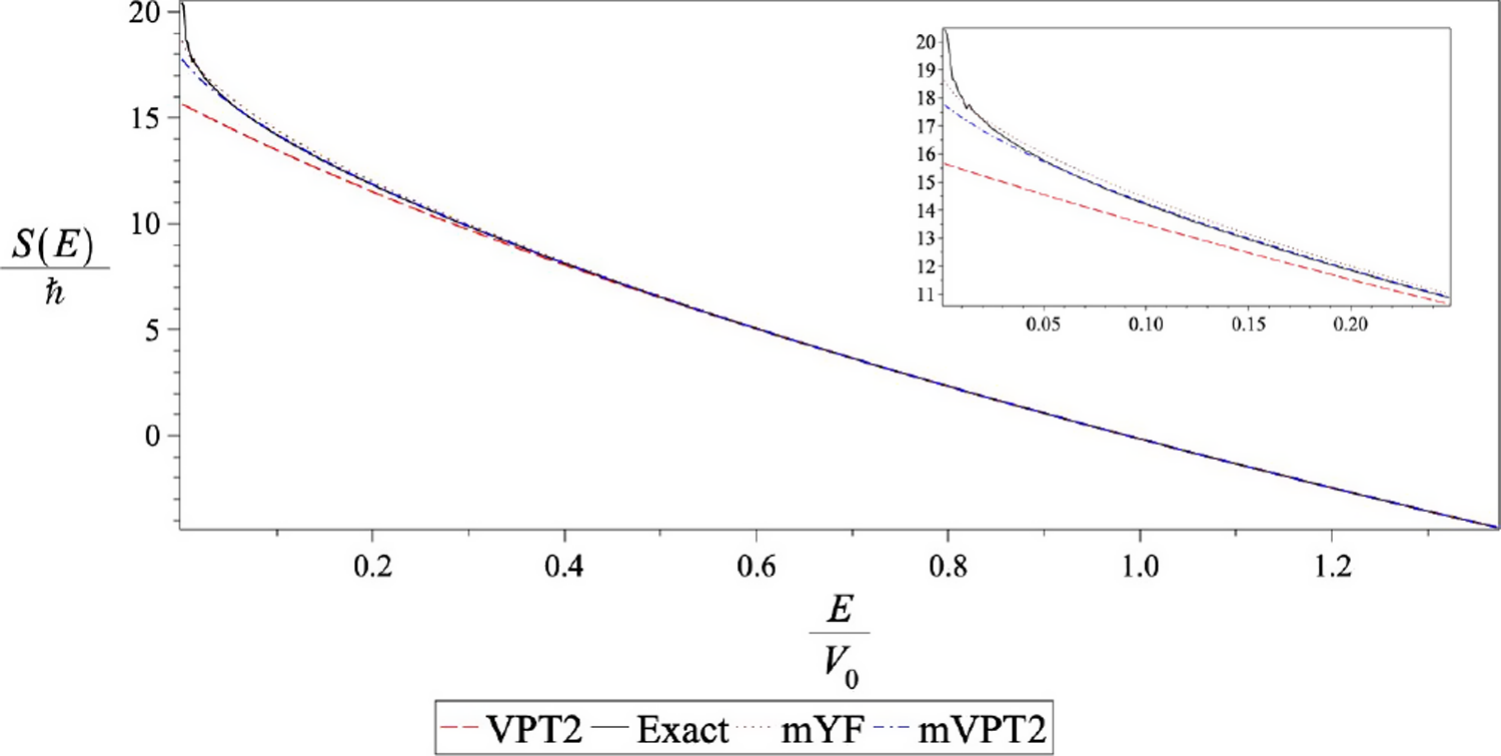
Energy dependence of actions on the Gaussian barrier. The solid line is the exact action obtained, as defined in eq 1.3. The dashed line (red) shows the standard VPT2 action. The dash-dotted line (blue) depicts the modified VPT2 action and the dotted line shows the modified YF action. The inset plot magnifies the differences at low energies.

A qualitative understanding of these results can be obtained by comparing the energy dependence of the various actions used, as presented in Figure 1. At high energies above the barrier energy, they are all very similar. As one goes into the deep tunneling regime, one observes that the VPT2 action is significantly smaller than the exact, mVPT2 and mYF actions. This is why the VPT2 theory fails in the low-temperature limit, giving a transmission factor that is too large. The dependence of the VPT2 action on the energy is not accurate enough. The mVPT2 action is closer to the exact action than the mYF counterpart and therefore gives a better estimate for the thermal transmission factor. The mVPT2 action is somewhat lower than the exact action so it overestimates the transmission factor while the mYF theory is slightly above the exact action, underestimating the exact thermal result.

### 3.4 Tanh Barrier

**Figure 2:**
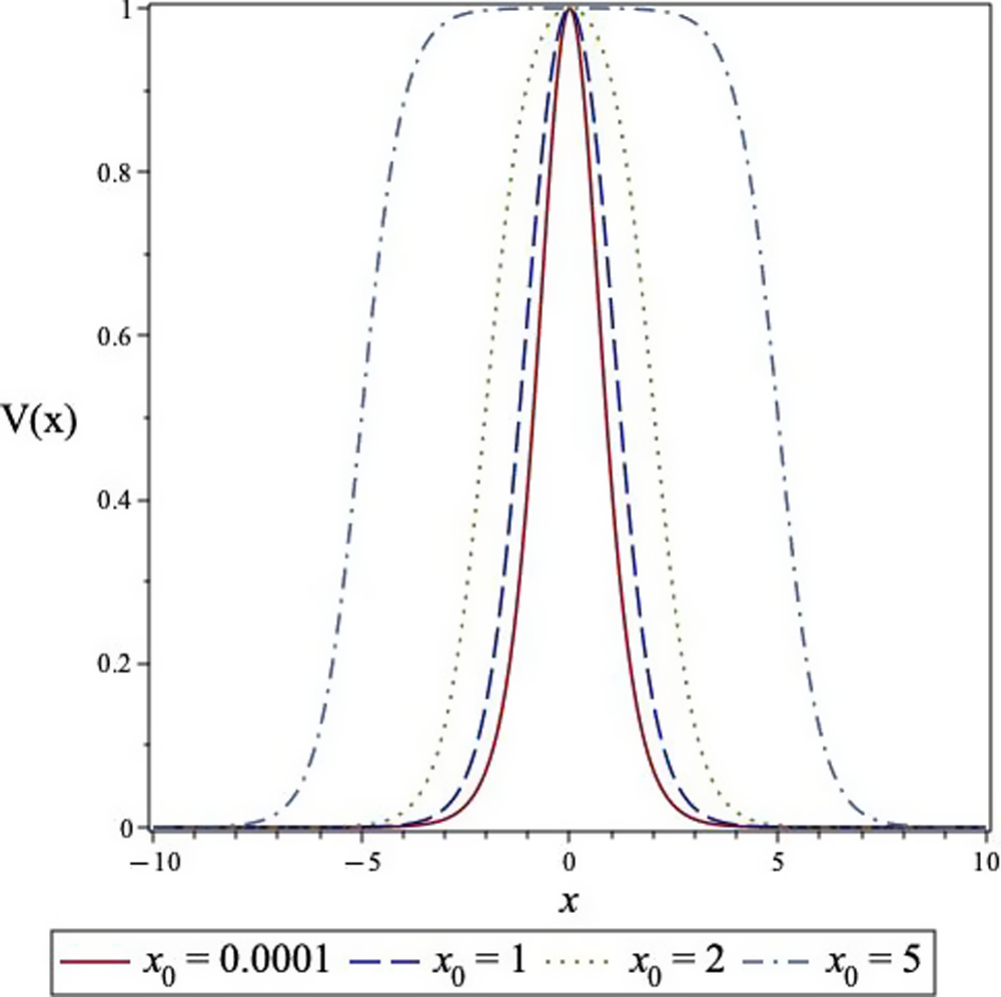
Variation of the tanh potential with width parameter *x*_0_.

The symmetric tanh barrier is defined as
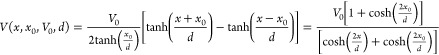
3.17Here *V*_0_ is the barrier height and *d* is the length parameter. We chose to study this case due to its dependence on the “width” *x*_0_ as visualized in Figure 2. The tanh potential is plotted for various values of *x*_0_ (*d* = 1.0). In the limit of *x*_0_ → 0, the potential is identical to the symmetric Eckart barrier. When *x*_0_ becomes large, the potential tends to a step potential with a total width of 2*x*_0_.

The second and fourth derivatives of the tanh potential at the barrier top (*x*^‡^ = 0) are
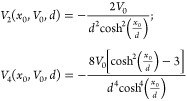
3.18 One notes that although *V*_2_ is negative for all values of *x*_0_, it becomes exponentially small as *x*_0_ increases. At the same time, the fourth derivative changes sign when . The ratio of the fourth derivative to the second derivative remains finite for all values of *x*_0_ even when *V*_2_ vanishes in the limit *x*_0_ → *∞*. The reason we chose to study this potential is the change of sign of *V*_4_. When it is negative, the energy shift *E*_0_ in the mVPT2 theory and action shift Δ*S* in the mYF theory become positive so that the half point comes at an energy that is higher than the barrier energy. To see the implications of this we consider 2 cases of the tanh potential, the first case is when *x*_0_ = 1.0 and therefore *x*_FF_ < 0; *E*_0_ < 0. The second case is when *x*_0_ = 2.0 so that *x*_FF_ > 0; *E*_0_ > 0.

#### 3.4.1 *x*_0_ = 1.0

In this case, we have *x*_FF_ < 0 so that the mVPT2 and mYF energy-dependent transmission coefficients are obtained from eqs 2.15 and 2.23, respectively. The Euclidean action below the barrier is computed numerically using the same algorithm as that used in the Gaussian potential. The numerically exact energy-dependent transmission coefficients are computed using the same program for the solution of the Schrödinger equation as the one used for the Gaussian barrier with an error tolerance of ∼4 × 10^–5^. All computations are obtained by setting *ℏ* = 1.0, *m* = 1.0, *V*_0_ = 1.0, *d* = 1.0. With these parameters, we find that the energy shift *E*_0_ = −0.0162 and the action shifts are Δ*S* = −0.1114 and *S*_VPT2_^*^ = 0.000143. As in the Gaussian barrier, the magnitude of *S*_VPT2_^*^ is much smaller than Δ*S*, so that it is essentially negligible. The results shown in Table 4 are calculated for various inverse temperatures *u*_e_ = *ℏ*βω_e_^‡^, where ω_e_^‡^ is the barrier frequency for the Eckart barrier (*x*_0_ = 0). We note though that the “true” value of *u*, using the actual barrier frequency of the tanh potential, is much smaller due to the small absolute value of the second derivative at the barrier.

**Table 4:** Thermal Transmission Coefficients for the Tanh Barrier (*x*_0_ = 1.0)

*u*_e_ = *ℏ*βω_e_^‡^	*T*_exact_	*T*_mYF_	*T*_mVPT2_	*T*_VPT2_	1 + κ_2_	1 + κ_2_ + κ_4_
0.5	1.013	1.007	1.007	1.007	1.008	1.013
1.0	1.036	1.023	1.023	1.024	1.025	1.036
1.5	1.070	1.048	1.048	1.051	1.051	1.070
2	1.114	1.081	1.083	1.087	1.085	1.116
3	1.242	1.180	1.183	1.195	1.180	1.247
4	1.435	1.331	1.335	1.361	1.311	1.441
6	2.131	1.870	1.884	1.985	1.676	2.094
8	3.649	3.021	3.063	3.442	2.181	3.277
10	7.179	5.630	5.766	7.179	2.826	5.274
12	16.067	12.060	12.511	17.846	3.612	8.452
14	40.210	29.284	30.845	51.229	4.537	13.259
16	110.277	78.964	84.553	163.379	5.602	20.227
18	325.058	231.379	251.914	559.887	6.807	29.969
20	1013.079	722.585	799.723	2013.131	8.153	43.182

Table 4 shows the thermal transmission coefficients obtained by using the various estimates. Remarkably, although the VPT2, mYF, and mVPT2 results are the same in the high-temperature limit, they are noticeably smaller than the exact result. This may be understood from the rightmost columns where we tabulate the thermal transmission coefficient obtained with the exact transmission coefficient containing terms of up to order *ℏ*^2^ (1 + κ_2_) and up to order *ℏ*^4^ (1 + κ_2_ + κ_4_). κ_2_ is given in eq 2.3, κ_4_ for symmetric potentials is^37^
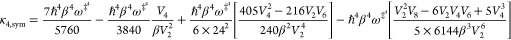
3.19

**Figure 3:**
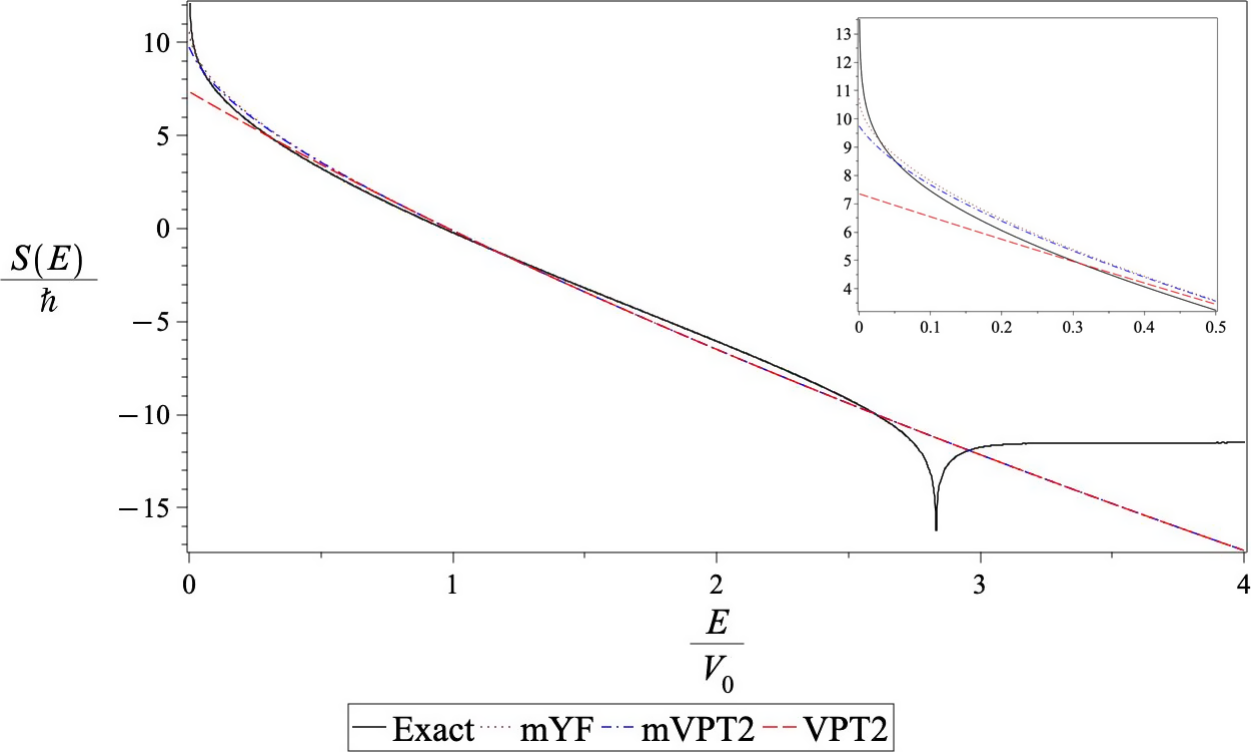
Energy dependence of the action for the tanh (*x*_0_ = 1.0) barrier. The solid line is the exact action (eq 1.3), the dashed line (red) shows the standard VPT2 action (eq 2.11), the dash-dotted line (blue) is the modified VPT2 action, and the dotted line shows the modified YF action as functions of the energy. The inset plot magnifies the differences between the various estimates at low energy.

Inspection of Table 4 shows that at high temperatures there is good agreement between the VPT2, mYF, and mVPT2 approximations and 1 + κ_2_ but not with the exact result. κ_4_ is not negligible compared with κ_2_ so that only when including the κ_4_ term does one get good agreement with the exact result. The VPT2, mYF, and mVPT2 expressions have not been constructed in a way that leads to the exact κ_4_ term. In this tanh potential, the fourth-order term is significant due to the low absolute value of the second derivative. As the temperature is reduced, leading to the deep tunneling regime, the mVPT2 and mYF results are in fair agreement with the exact results; at *u*_e_ = 20 the respective errors are ∼21 and ∼29%. The VPT2 results are, as in the previous cases, much too high again reflecting the incorrect quadratic dependence of the energy on the action at low energies, which, as shown in Figure 3 is corrected in the mVPT2 and the mYF theories, which are based on the numerical Euclidean action.

Figure 3 which shows the energy dependence of the actions accentuates a property of the tanh potential, namely, that there are resonant features when the energy is above the barrier energy. This is the source of the dip in the exact action when *E* ∼ 2.8. None of the approximate theories can replicate this effect, but it is not very important when considering the thermal transmission coefficient, which washes out the resonance feature.

#### 3.4.2 *x*_0_ = 2.0

**Figure 4:**
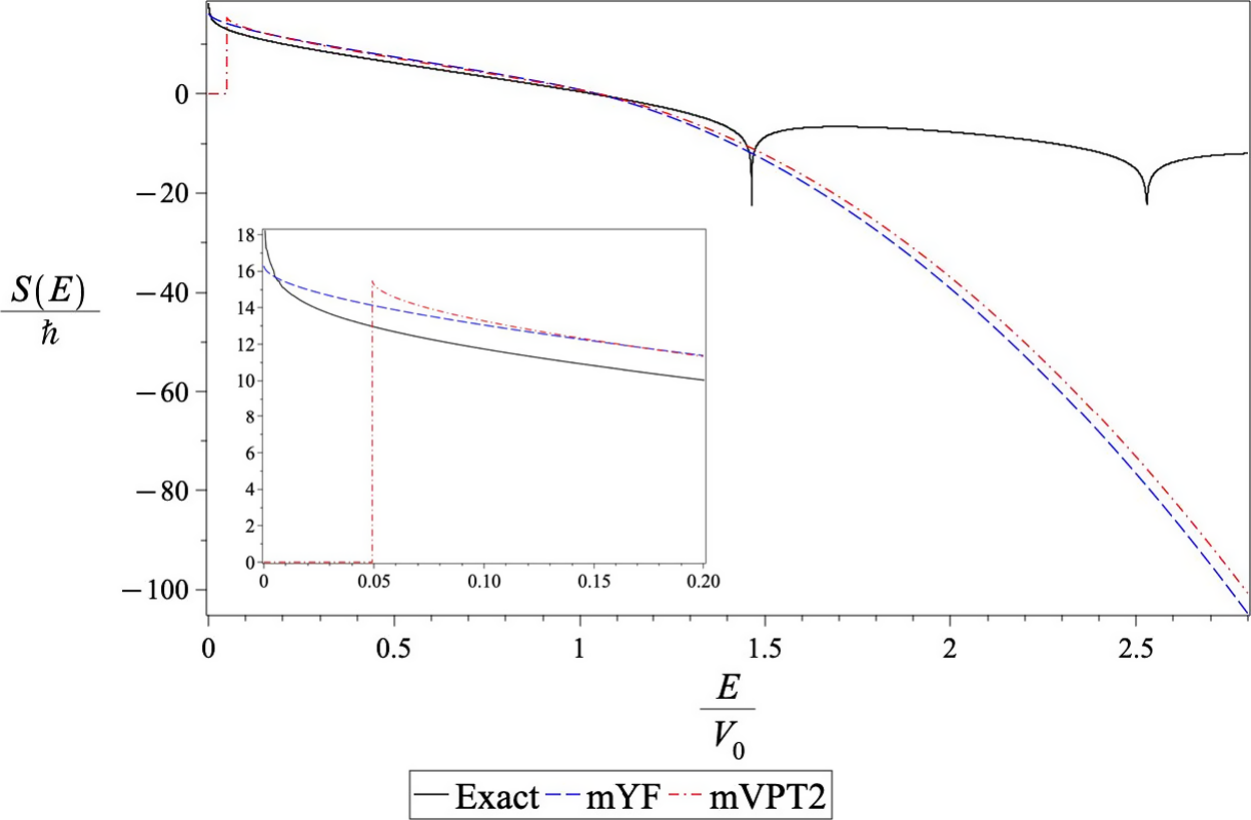
Energy dependence of the action for the tanh (*x*_0_ = 2.0) barrier. The solid line is the exact action as obtained from eq 1.3. The dash-dotted line (red) depicts the modified VPT2 action, and the dashed (blue) line shows the modified YF action. The inset shows the deviations of the various types of action at low energies.

In this case, as already mentioned, due to the negative fourth-order derivative, *E*_0_ and *x*_FF_ are positive. The half point is above the barrier energy. As a result, the VPT2 theory is not valid and one must use the above barrier action as in eqs 2.16 and 2.25 for the mVPT2 and mYF transmission coefficients, respectively. The thermal rates are calculated using the units *ℏ* = 1.0, *m* = 1.0, *V*_0_ = 1.0, and *d* = 1.0. With these, one has *E*_0_ = 0.0493 and Δ*S* = 0.8233, and both are positive. The resulting actions are compared in Figure 4. Here, not only are the above barrier resonance features in the exact action accentuated but one sees that the Gaussian approximation for the action above the barrier energy has a magnitude that grows much too rapidly with increasing energy. The mVPT2 action is less negative than the mYF action, implying that *T*_mVPT2_ < *T*_mYF_. As one goes down in energy, one encounters the difficulty with the mVPT2 theory, discussed in the previous section. Since *E*_0_ is positive, the action becomes ill-defined at energies that are lower than *E*_0_. This problem does not arise in the mYF expression.

Inspection of the data presented in Table 5 is instructive. At high energy, the thermal transmission coefficient is less than unity, quantum reflection becomes important, and this is captured by both the mYF and mVPT2 theories. As in the case of *x*_0_ = 1, at high temperatures, the κ_4_ term is important and is not captured by the two approximations. As the temperature is decreased, the quality of both the mVPT2 and mYF estimates deteriorates. At the lowest temperature considered (*u*_e_ = 80), the mYF estimate is a factor of ∼2.35 too small. At higher temperatures, i.e. *u*_e_ = 28, the error is even larger, the exact result is a factor of ∼3.5 larger than the mYF estimate.

**Table 5:** Thermal Transmission Coefficients for the Tanh Barrier (*x*_0_ = 2.0)

*u*_e_ = *ℏ*βω_e_^‡^	*T*_exact_	*T*_mYF_	*T*_mVPT2_	1 + κ_2_	1 + κ_2_ + κ_4_
0.5	0.993	0.988	0.987	0.989	0.991
1.0	0.987	0.978	0.977	0.980	0.984
1.5	0.984	0.968	0.967	0.972	0.980
2	0.983	0.961	0.960	0.965	0.979
3	0.991	0.950	0.950	0.957	0.986
4	1.010	0.945	0.947	0.954	1.004
6	1.090	0.956	0.966	0.967	1.080
8	1.245	1.004	1.026	1.003	1.214
12	2.113	1.301	1.388	1.145	1.702
16	5.860	2.517	2.806	1.382	2.575
20	29.448	9.501	10.347	1.713	3.981
24	232.606	67.298	-	2.138	6.104
28	2369.450	675.931	-	2.658	9.164
32	27,721.188	8047.932	-	3.271	13.418
36	3.522 × 10^5^	1.051 × 10^5^	-	3.979	19.162
44	6.576 × 10^7^	2.099 × 10^7^	-	5.678	36.488
52	1.376 × 10^10^	4.705 × 10^9^	-	7.753	64.249
60	3.082 × 10^12^	1.126 × 10^12^	-	10.205	106.149
68	7.216 × 10^14^	2.807 × 10^14^	-	13.033	166.489
80	2.732 × 10^18^	1.161 × 10^18^	-	17.983	302.446

**Figure 5:**
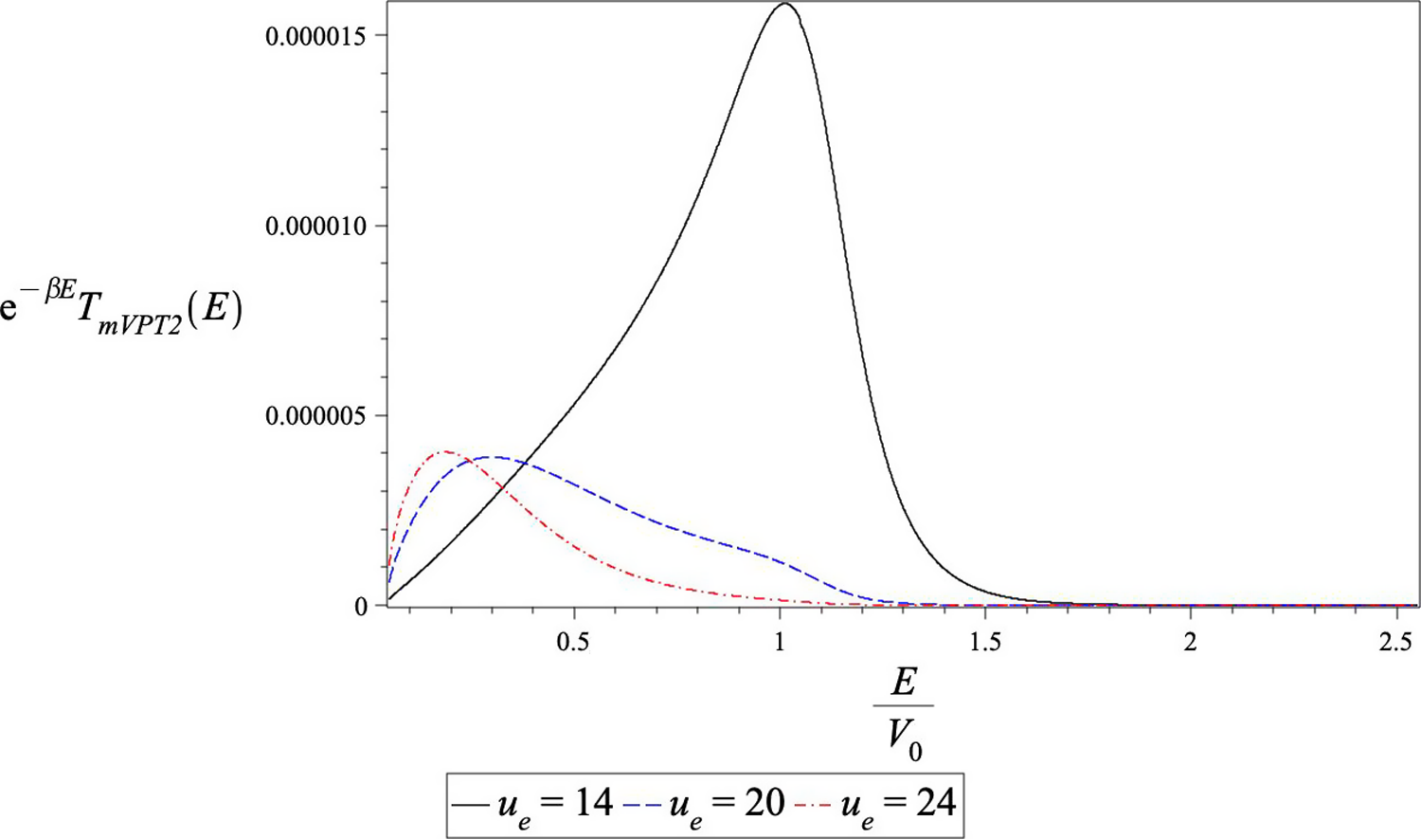
Variation of the *T*_mVPT2_ integrand with energy for various inverse temperatures for the tanh *x*_0_ = 2.0 barrier. Note that the low energy part of the integrand is noticeably incomplete for *u*_e_ = 24. The *u*_e_ = 20 and *u*_e_ = 24 plots have been multiplied by a factor of 5 and 10, respectively.

The mVPT2 results are also problematic. In Figure 5, we plot the integrand of the thermal transmission coefficient for three different inverse temperatures, *u*_e_ = 14, 20, 24. One sees from the figure that up to *u*_e_ = 20 the integrand almost goes to 0 at low energy, however for *u*_e_ = 24 it does not and the error involved is no longer negligible. For this reason, Table 5 has the mVPT2 data only up to *u*_e_ = 20. One may come up with different suggestions for extrapolation of the mVPT2 transmission probability to *E* = 0, but the result would depend on the choice and, without a “good theory” would not really reflect on the quality of the result. We do note that in the range of validity, the mVPT2 results are a bit better than the mYF results. This case shows that the theory is incomplete when considering thick barriers. Fortunately, all “real” cases that we are aware of have positive fourth-order derivatives for the reaction coordinate at the barrier, so that these problems do not arise.^40^

**Figure 6:**
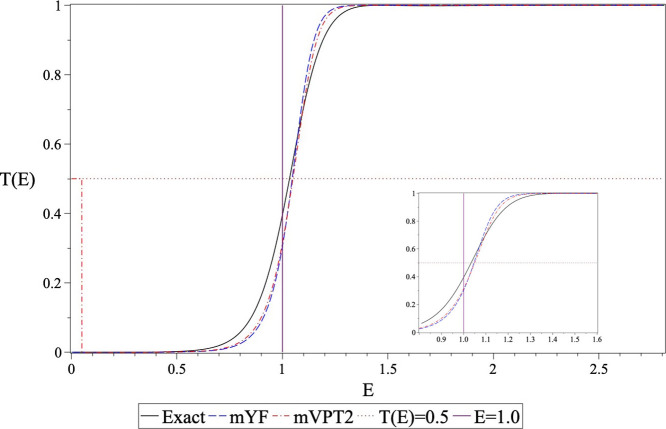
Energy dependence of the transmission probability for the tanh (*x*_0_ = 2.0) barrier. The solid (black) line shows the numerically exact transmission coefficient, the dashed (blue) line shows the mYF result, and the dash-dotted (red) line shows the mVPT2 result. The dotted lines accentuate that the half point in this case is higher than the barrier energy and that the mVPT2 and mYF approximations predict it rather accurately.

Even for this extreme case, there is a redeeming feature. The magnitudes of *E*_0_ and Δ*S* do reflect almost quantitatively the half point of the transmission probability, which is greater than the barrier height. This is shown in Figure 6 where we compare the numerically exact energy-dependent transmission probability (solid black line) with the mVPT2 (dash-dotted red line) and the YF (dashed blue line) approximations. As may be seen, at energies below the barrier height, the differences between the approximate theories and the exact results are noticeable; however, at the half point, they come rather close to each other.

## 4 Multidimensional Generalization

### 4.1 Review of Miller’s Uniform Theory

We assume a Hamiltonian with *N* + 1 degree of freedom
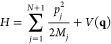
4.1and that the potential is characterized by a saddle point, at the point **q**^‡^, with energy *V*^‡^, with *N* stable directions *j* = 1,..., *N* with frequencies ω_*j*_^‡^. The *N* + 1-th degree of freedom which is unstable has an imaginary frequency ω^‡^.

We further assume that at energies *E* < *V*^‡^ there exists a periodic orbit (instanton) on the inverted potential energy with period , Euclidean action
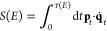
4.2and *N* stability frequencies ω_*j*_(*E*). Following Miller,^41^ a thermal cumulative transmission probability is defined as
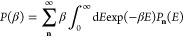
4.3where the summation is over all stable modes of the transmission probability
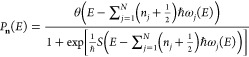
4.4where θ(*x*) is the unit step function.

Changing variables from *E* to  and ignoring the fact that the stability frequencies are energy-dependent, performing the summation over all stable mode states gives the result
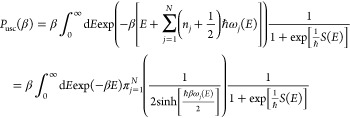
4.5where the second line is obtained after summation over the vibrational quantum numbers. The remaining energy integral may be estimated analytically using the steepest descent approximation, but this is not of interest at this point.

### 4.2 Brief Review of VPT2 Theory

A central feature of VPT2 theory is that the action of the unstable orbit, whether the energy is above or below the barrier height, is obtained from a quadratic expansion of the energy about the saddle point energy in terms of the action of the orbit, which is obtained from quantum second-order perturbation theory:
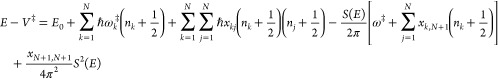
4.6Here the frequencies ω_*k*_^‡^ are the stable normal-mode frequencies at the saddle point, the anharmonic coefficients are given in terms of higher-order derivatives of the potential at the saddle point, as given in detail in ref (42). The central difference between this expression and the one used in ref (20) is the addition of the zero point energy term *E*_0_ which in the multidimensional case depends on the expansion coefficients of the potential up to the fourth order about the saddle point.

Using the notation
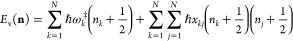
4.7for the energy in the stable modes, the quadratic energy action relationship may be inverted such that
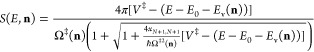
4.8where the “effective barrier frequency” is
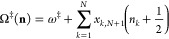
4.9

The thermal cumulative transmission probability at energy *E* is given in eq 4.3. Changing variables as before from *E* to ε = *E* – *E*_0_ – *E*_v_(**n**) allows us to write the VPT2 expression as 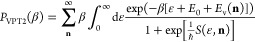
4.10Due to the quadratic terms, here, it is no longer possible to carry out the summation over the stable modes analytically as in eq 4.5. In practice, summation is implemented numerically. With this formulation, the only dependence left in the action on the internal energy is through the effective barrier frequency Ω^‡^(**n**).

### 4.3 Multidimensional mVPT2 and mYF Theories

As we saw in the previous subsection, in the multidimensional case, the energy is shifted not only by the zero point energy through *E*_0_ but also through the vibrational energy of the stable modes *E*_v_(**n**). To generalize the one-dimensional mVPT2 theory we rewrite the thermal probability as
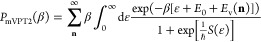
4.11where the action *S*(ε) is the energy-dependent action of the instanton and is not forced to be the quadratic action as in eq 4.8. If one ignores the quadratic part in *E*_v_(**n**) one recovers Miller’s result as given in eq 4.5 with two differences. One is the addition of the zero-point energy shift *E*_0_. The other is that the frequencies of the stable modes ω_*j*_^‡^ are the stable normal-mode frequencies at the saddle point, and not the stability frequencies of the instanton. However, by ignoring the latter difference and shifting the energy by *E*_0_ we obtain a “modified Miller P” or in short, mMP theory, to indicate that it is related to the perturbation theory derived energy shift:
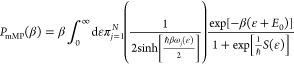
4.12and one notes that this is the natural generalization of eq A.3 in the one-dimensional case.

Similarly, one may write down a modified YF theory by adding a constant term to the action as in eq 2.20 such that
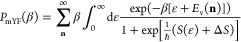
4.13and the analogous expression which we call “modified Miller Y” will be
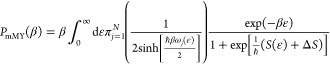
4.14The energy integration in all of these expressions may be estimated using the steepest descents as in ref (34).

## 5 Discussion

The central result of this paper is that due to the known exact *ℏ*^2^ term of the quantum thermal transmission coefficient through a barrier, one can modify the uniform semiclassical expression so that it too is exact in this limit using two different methods, both of which lead to significant improvement to the semiclassical theory. One way is to shift the energy by the known zero point energy shift obtained by the second-order perturbation theory. This modifies the VPT2 rate theory so that on the one hand, it remains unchanged in the moderate to high-temperature limit but on the other hand, it is much improved in the low-temperature limit. This gives us the mVPT2 theory that is applicable at all temperatures; the only proviso is that the fourth-order derivative at the barrier is positive. Our experience thus far is that in most molecular reactive systems this is the case.^40^ Is such a theory more precise than CMD or RPMD? Numerical tests on multidimensional systems are needed.

Our second suggestion is to shift the Euclidean action by a constant. Such a theory is also implementable in many dimensions at all temperatures. It has the advantage over the mVPT2 theory in that it is valid at all energies, irrespective of the sign of the fourth derivative. For the one-dimensional Eckart potentials, it is the most accurate semiclassical-based theory available.

One of the critiques of approximate methods such as CMD and RPMD is that they do not have objective criteria such as a leading order correction term to assess their accuracy. At this point, this is also true for the modified semiclassical theories presented here. However, at least in principle, we know the exact 4-th order term, of order *ℏ*^4^ and so one could further modify the uniform semiclassical theory to be exact to the same order and this added term could give an objective estimate as to the accuracy of the theory. However, there would be a hefty price to pay, and that is, for the fourth-order term, one needs up to the eighth-order derivative of the potential at the barrier, and it is doubtful that present ab initio methods are accurate enough for this purpose.

We outlined how the modified one-dimensional theory presented here may be incorporated within a multidimensional theory. One may expect that the implementation of the modified VPT2 theory, as given in eq 4.11 should be straightforward. For energies above the crossover temperature, one would employ the “standard” MVPT2 theory. For energies below, it would be necessary to generate the energy-dependent instanton trajectory and then average it over energy and vibrational states. The sum over vibrational states has been implemented efficiently within the VPT2 theory,^22^ and “fast” algorithms for locating the energy-dependent instanton orbit are also available.^25,26,33^ In addition, the energy integration may be implemented using the steepest descent algorithm as described in ref (32). The difference between the vibrational perturbation theory-based algorithm and the Yasumori algorithm is small; therefore, the same methods may be used for the latter case. The real question is how much of an improvement these methods are as compared to “standard” VPT2 theory. This will be explored in future work.

The theories presented here also impact the uniform thermal instanton method. As is well understood, the instanton expression is obtained by a steepest descent estimate of the energy integration inherent to the thermal transmission factor. The uniform semiclassical steepest descent condition is^34^
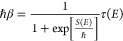
5.1where τ(*E*) is the period of the instanton orbit. In the VPT2 theory, this condition changes to
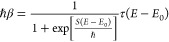
5.2and in the mYF theory, it is
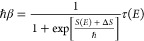
5.3If, as is usually the case, *E*_0_ and Δ*S* are negative, this implies that the crossover temperature, that is the temperature at which the instanton energy is the same as the barrier height is slightly lower than *ℏ*βω^‡^ = π as would be obtained in the usc expression. Another aspect is that in the usc theory, all that one needs as input is the instanton orbit and its stability frequencies. The present modified theories are more expensive to implement; one needs to evaluate the higher-order derivatives of the potential at the saddle point.

VPT2 theory would seem to be a heuristic theory since it involves a replacement of a term such as *ℏ*ω (*n* + 1/2) with the imaginary classical Euclidean action ω^‡^*S*(*E*). Although highly suggestive, there is no proof for this. A central result of this paper is that the zero point energy shift obtained from second-order perturbation theory is the same shift that affects the uniform semiclassical expression for the energy-dependent transmission coefficient and that the result obtained from the replacement is exact to order *ℏ*^2^. This implies a more rigorous justification for the mVPT2 theory.

## Appendix

In this Appendix, we derive in detail the expansion of the thermal transmission coefficient up to second order in *ℏ* using a generalized form of the uniform semiclassical transmission coefficient:

A.1

Using the quadratic expansion for the action as in eq 2.4, introducing the dimensionless parameters

A.2

and changing variables from *E* to *Y* = β [*V*^‡^ – (*E* – *E*_0_)] /α allows us to rewrite the expression for the transmission coefficient κ_u_(β) as

A.3

Since we are considering the limit of high temperature or equivalently small α we may replace the lower limit with –*∞* with a negligible exponentially small error. We then use the expansion

so that

A.4

Expanding further

implies that to leading order

A.5

with

A.6

and

A.7

We then perform each integral separately. First, we note^43^ that

A.8

Then, because

A.9

we may integrate by parts to rewrite the second integral as

A.10

We are then interested in expanding the transmission coefficient up to second order in α. We assume that both constants Δ*S* and Δ*E* are of the order of α^2^:

A.11

A.12

so that after some tedious algebra (using Maple) we find the central result of this Appendix

A.13
